# Laser-Induced Graphene on Polyimide: Material Characterization Toward Strain-Sensing Applications

**DOI:** 10.3390/s25247641

**Published:** 2025-12-17

**Authors:** Yessenia Ibeth Paucar, Fernando Pantoja-Suárez, Enric Bertran-Serra, Fernando Sánchez, Katherine Moreno

**Affiliations:** 1 Materials Department, Faculty of Mechanical Engineering, Escuela Politécnica Nacional, Ladrón de Guevara E11-253, P.O. Box 17-01-2759, Quito 170525, Ecuador; fernando.pantoja@epn.edu.ec (F.P.-S.); fernando.sanchez01@epn.edu.ec (F.S.); 2 ENPHOCAMAT (FEMAN) Group, Deparment of Applied Physics, Universitat de Barcelona, Martí i Franquês 1, 8028 Barcelona, Spain; ebertran@ub.edu; 3 Institute of Nanoscience and Nanotechnology (IN2UB), Universitat de Barcelona, 08028 Barcelona, Spain; 4 Department of Extractive Metallurgy, Escuela Politécnica Nacional, Ladrón de Guevara E11-253, P.O. Box 17-01-2759, Quito 170525, Ecuador; katherine.moreno@epn.edu.ec

**Keywords:** bending, gauge factor, graphene, laser diode, Raman, SEM, UV

## Abstract

**Highlights:**

**What are the main findings?**

- Low-power laser scribing of polyimide produced porous graphene-like conductive patterns suitable for strain sensing. Laser power was the most critical parameter, yielding sheet resistances down to ~20 Ω sq^−1^ and a gauge factor up to ~1.1.
- Increasing the number of laser passes (multiple engravings) deteriorated graphene material quality and significantly reduced the strain gauge factor of the sensor.

**What is the implication of the main finding?**

- A 450 nm diode laser can be used to fabricate low-cost, flexible graphene strain sensors without chemical processes. This approach enables accessible production of strain gauges on polymer substrates for flexible electronics and structural health monitoring.
- The feasibility demonstrated here suggests that inexpensive diode lasers could be deployed for on-demand graphene-based sensor fabrication, potentially accelerating research and applications in wearable and flexible sensor technology.

**Abstract:**

This study investigates the effect of laser power, focal length, and number of passes on the fabrication of graphene-based strain sensors using a 450 nm diode laser at the upper limit of the UV spectrum. Polyimide substrates were irradiated to produce laser-induced graphene, and the resulting sensors were evaluated under three-point bending tests. The main requirements for deformation sensors—adequate conductivity, mechanical stability under bending, and high sensitivity (gauge factor)—were assessed through morphological analysis by SEM, Raman spectroscopy, and electrical characterization using the Van der Pauw method. The results indicate that laser power is the critical factor influencing graphene quality and sensor performance, while focal length has a negligible effect and additional passes reduce material quality and sensitivity. Overall, this work demonstrates the feasibility of producing functional, low-cost graphene strain sensors with a commercial diode laser, offering a scalable and affordable alternative for sensor fabrication.

## 1. Introduction

The study of materials and the development of novel ones represent a fundamental pillar of engineering and, therefore, of technological progress. Since its discovery in 2004, graphene has become a reference material among carbon allotropes such as graphite [1] and diamond, owing to its unique nanometric hexagonal structure. This remarkable material has demonstrated extraordinary physical and chemical properties that translate into applications including energy storage, high-performance sensors, coatings, thermal management, pollutant adsorption, water desalination, and biomedical devices [2,3]. Its exceptionally high electrical conductivity, combined with mechanical robustness and low density, makes graphene particularly attractive for lightweight and efficient electronic devices.

Within the broad field of graphene research, laser-induced graphene (LIG) has gained increasing attention. A bibliometric analysis of 87 articles indexed in Scopus revealed that the most frequent keywords are “graphene,” “laser-induced graphene,” and “portable sensors,” often associated with “deformation.” The co-occurrence clusters emphasize themes of graphene and characterization, strain gauge and sensors, applications, and polymers/precursors. The annual production of articles has grown steadily, with a temporary decrease in 2023, although 12 articles had already been published in the first half of that year. In terms of geographic distribution, China leads scientific output, followed by South Korea, the United States, and Japan. Significantly, no Latin American countries appear among the leading contributors, underscoring the importance of developing research in this region. Highlighting this disparity emphasizes the need for accessible methods such as diode-laser graphene synthesis, which can democratize entry into this technological field.

In the context of applications, sensors are among the devices that benefit most from graphene. Deformation and chemical detection sensors are especially promising [4]. However, conventional methods to obtain high-quality graphene remain relatively costly [5] and complex compared to other widely used materials such as silicon [6], copper [7], or lithium [8]. Consequently, early graphene research was largely concentrated in well-funded institutions and countries. More recently, the focus has shifted toward developing safer, eco-friendly, and cost-effective production routes [9].

Among these emerging techniques, LIG stands out as a scalable and relatively low-cost process. First reported in 2014 by Lin et al. [10], LIG was obtained by irradiating polymeric substrates with an infrared CO_2_ laser, producing porous three-dimensional graphene networks. Since then, CO_2_-laser-based LIG has been extensively reported, yielding high-quality strain and chemical sensors [11]. Nonetheless, studies on graphene synthesis using low-cost diode lasers remain scarce. This gap represents a barrier to the broader dissemination of graphene-based technologies and calls for systematic research to validate whether commercially available diode lasers can produce functional sensing materials.

This study addresses that challenge by demonstrating the feasibility of producing LIG on polyimide substrates using a 450 nm commercial diode laser. The experimental design considers laser power, number of passes, and focal distance as independent variables, while the strain gauge factor serves as the dependent variable. Pre-tests were performed to optimize sensor geometry based on electrical resistance before proceeding to characterization. The resulting LIG samples were analyzed using SEM and Raman spectroscopy, while electrical sheet resistance was determined with the Van der Pauw method. Finally, the fabricated sensors were integrated into polypropylene specimens following ASTM D790 standards [12], and their strain-sensing performance was assessed under three-point bending conditions.

In summary, this work aims to demonstrate that a low-power diode laser can effectively induce graphene formation for the fabrication of functional strain sensors. Beyond establishing the processing–structure–property relationships, the study highlights the potential of this environmentally friendly and cost-effective technique for scaling electronic devices based on graphene, with a particular emphasis on expanding access to such technologies in resource-limited settings.

Therefore, the objective of this study is to demonstrate the feasibility of fabricating laser-induced graphene (LIG) using a low-power commercial diode laser (450 nm) as a sustainable and cost-effective alternative to conventional high-power methods. Specifically, the effects of laser power, focal distance, and number of passes on the microstructure, sheet resistance, and gauge factor of LIG were systematically investigated. The study also seeks to evaluate the potential application of these LIG structures as strain-sensing elements, providing a pathway toward low-cost, flexible, and scalable sensors for applications in structural health monitoring, wearable electronics, and flexible devices.

## 2. Materials and Methods

### 2.1. Materials and Equipment

**Substrate:** Kapton^®^ HN polyimide film (DuPont, USA), thickness 75 µm.

**Contacts:** PELCO^®^ Colloidal Silver Ink (Ted Pella Inc., USA); 3M™ copper foil tape (3M, USA).

**Adhesives:** Loctite^®^ cyanoacrylate (Henkel, Germany).

**Support beams:** Commercial polypropylene specimens (Quito, Ecuador).

**Laser processing system:**

- CNC laser engraver Ortur LU2-4 (Ortur, China) with LU2-4 diode module, wavelength 450 nm, peak output ≈ 5 W.
- Controlled by GRBL firmware; scanning speed set at 1000 mm·min^−1^.
- Spot size at focal plane ≈ 0.2 mm.

**Environmental monitoring:**

- Digital thermohygrometer (China), accuracy ±2% RH and ±1 °C.

**Characterization instruments:**

- SEM: ASPEX PSEM Express (USA, 15 kV).
- FE-SEM: JEOL JSM-7100F (JEOL Ltd., Japan), up to 100,000× magnification.
- EDS: Oxford Instruments (UK) integrated in FE-SEM.
- Raman: Horiba LabRAM Evolution (Horiba Scientific, France), 532 nm laser (50 mW, filtered to 10%).
- Power supply: Keysight E36100A (Keysight Technologies, USA), accuracy ±0.05%.
- Multimeter: Keysight 34461A (Keysight Technologies, USA), accuracy ±0.01%.
- DAQ: National Instruments USB-6009 (NI, USA).
- Mechanical tests: Universal testing machine Tinius Olsen H25KS (USA), load cell accuracy ±0.5%.

**Strain calculation (ASTM D790):**

Flexural strain during three-point bending was determined using Equation (1) using the ASTM D790 formula:

ε = (6Dh)/L^2^ (1)

where *D* is the mid-span deflection (mm), *h* is the specimen thickness (mm), and *L* is the span length (mm).

### 2.2. Experimental Design

A mixed-level factorial design of experiments (DOE) was implemented to evaluate the influence of laser parameters on the properties of the LIG strain sensors. Three independent variables were considered:

- Number of passes: 1 or 2;
- Laser power: 0.33, 0.44, and 0.55 W;
- Focal distance: 50 mm and 51 mm.

The complete set of experimental conditions is summarized in Table 1.

Preliminary experiments established the minimum power for carbonization. A value of 0.33 W was identified as the threshold for forming a continuous conductive path, while 0.55 W represented the maximum stable output of the diode laser. The scanning speed was fixed at 1000 mm·min^−1^, and the spot size at the focal plane was ≈0.2 mm. A focal distance of 50 mm provided the smallest spot (maximum fluence), whereas moving the head to 51 mm slightly defocused the beam, reducing fluence and enlarging the interaction area.

Each experimental condition generated one LIG sensor sample, resulting in a total of 12 samples. The response variables were:

- Sheet resistance (van der Pauw method).
- Microstructure and composition (SEM, EDS).
- Structural order (Raman spectroscopy).
- Strain sensitivity (gauge factor from bending tests).

**Figure 1:**
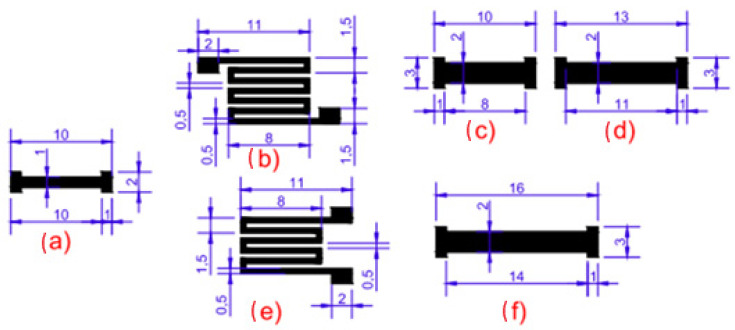
Geometry options for graphene sensors. Adapted and redrawn by the authors based on references [13,14,15,16,17,18,19].

Additionally, pre-tests compared six candidate sensor geometries laser-scribed on Kapton^®^ film. Their electrical resistances were measured, and the compact serpentine design (geometry c) exhibited the lowest resistance (~126.9 Ω) and minimal warping. This geometry, with a meandered conductive track of ≈0.25 mm width and pads for electrical connections, was selected for all subsequent experiments. The tested options are shown in Figure 1.

### 2.3. Fabrication of LIG Strain Sensors

Polyimide sheets (≈60 × 20 mm) were cleaned with 70% isopropyl alcohol. The optimized serpentine pattern was designed in CAD and converted into G-code for laser engraving. The diode laser was operated according to the DOE parameters; in two-pass conditions, the second scan was performed perpendicular to the first. After irradiation, the LIG appeared as a black porous track.

Electrical contacts were prepared by applying silver ink at both ends of the pattern, followed by copper tape for reinforcement. Sensors were bonded at mid-span to polypropylene beams using cyanoacrylate adhesive, and a protective tape was added to avoid delamination during bending.

### 2.4. Morphological and Chemical Characterization

The surface morphology of the LIG was analyzed using SEM and FE-SEM.

- Low magnification (200–5000×) was used to check pattern continuity and roughness.
- High magnification (up to 100,000×) revealed pore structure.
- Line spacing and overlap (~75–80%) were measured from SEM images.

Elemental composition was determined by EDS, confirming that the material was predominantly carbon (>96%) with trace oxygen.

### 2.5. Raman Spectroscopy

Raman spectroscopy was used to evaluate the structural quality of the LIG. Spectra were acquired with a 532 nm laser, recorded over 100–4000 cm^−1^. Ten random points were analyzed for each sample to account for inhomogeneity.

The main bands (D ≈ 1340 cm^−1^, G ≈ 1590 cm^−1^, 2D ≈ 2670 cm^−1^) were fitted with Lorentzian profiles. The intensity ratios I_D_/I_G_, I_2D_/I_G_ and I_D_/I_D′_ were calculated to assess disorder, number of layers, and defect type.

### 2.6. Electrical Characterization by the Van der Pauw Method

The sheet resistance was measured using the four-probe Van der Pauw technique.

- Four copper electrodes were placed at the corners of the LIG pattern using silver ink.
- A source-meter applied currents between 0.1–1 mA while voltages were recorded.
- The Van der Pauw equation was used to calculate the sheet resistance:

(2) $$e^{-\frac{\pi *R_{vertical}}{R_{s}}}+e^{-\frac{\pi *R_{horizontal}}{R_{s}}}=1\;$$

Eight current–voltage configurations were tested for each sample, and the average value was reported.

### 2.7. Bending Tests and Gauge Factor Determination

Strain sensing performance was evaluated by three-point bending tests (ASTM D790). Sensors were integrated into a Wheatstone bridge circuit, and the resistance change during bending was recorded with a data acquisition system.

Strain was calculated from beam theory, ensuring values < 5% to remain in the elastic regime. The gauge factor (GF) was obtained from the slope of normalized resistance change versus strain:

(3) $$GF=\frac{\left(R_{1}-R_{2}\right)/R_{O}}{\varepsilon_{1}-\varepsilon_{2}}=\frac{\Delta R/R_{o}}{\Delta \varepsilon }\;$$

Multiple loading–unloading cycles were conducted to verify repeatability and hysteresis.

### 2.8. Fluence Calculation

The laser fluence (energy per unit area) was estimated to correlate energy input with LIG properties. It was calculated as:

(4) $$F=P*\;\nu =P*\frac{t}{A}$$

where P is the measured laser power, t is the exposure time, and A is the irradiated area. This parameter was used to interpret trends in sheet resistance and gauge factor across the DOE.

In three-point bending tests, strain (ε) was calculated according to ASTM D790, which relates mid-span deflection to surface strain on the outer fibers of the specimen. Although strain is commonly associated with tensile testing, this standard provides the appropriate formulation for flexural strain, ensuring consistency with bending geometry rather than tensile strain calculations.

## 3. Results

### Laser-Induced Graphene Morphology and Composition

**Figure 2:**
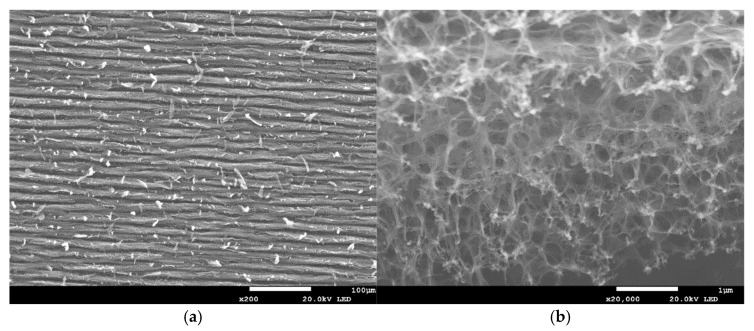
SEM micrographs of Sample 1. (**a**) Low-magnification view showing scan-line spacing across the patterned region. (**b**) High-magnification view highlighting micro/mesopores (~200–500 nm). Acquired with FE-SEM JSM-7100F (own data).

**Figure 3:**
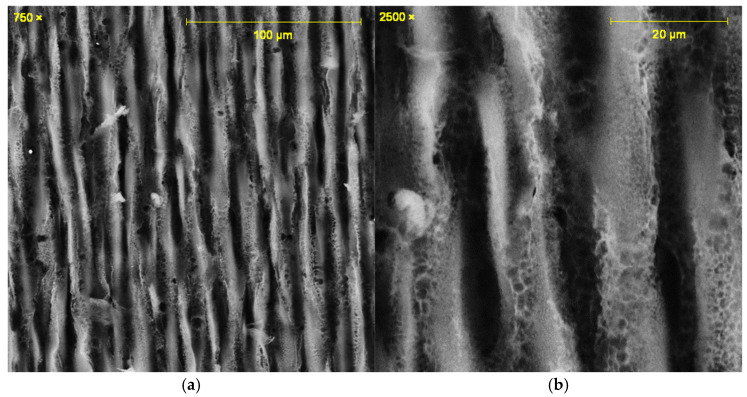
SEM micrographs of Sample 5. (**a**) Low-magnification view showing uniform scan-line distribution. (**b**) High-magnification view of pores (150–1600 nm). Acquired with ASPEX PSEM Express (own data).

**Figure 4:**
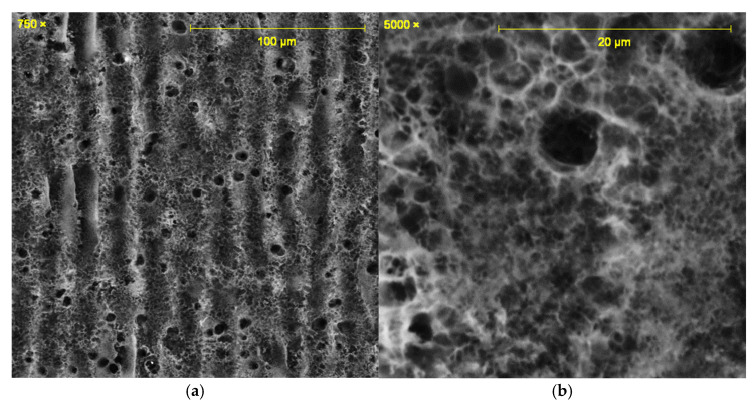
SEM micrographs of Sample 7. (**a**) Scan-line pattern with reduced spacing and uniform distribution. (**b**) Pore distribution (550–1400 nm). Acquired with ASPEX PSEM Express (own data).

**Figure 5:**
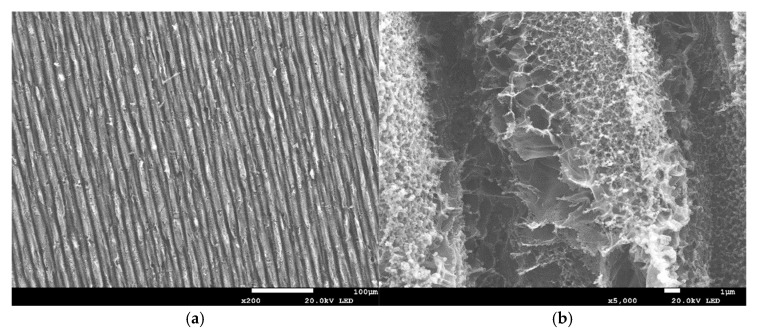
SEM micrographs of Sample 9. (**a**) Laser scan pattern showing well-defined tracks. (**b**) Pore distribution from 1500 to 140 nm. Acquired with FE-SEM JSM-7100F (own data).

**Figure 6:**
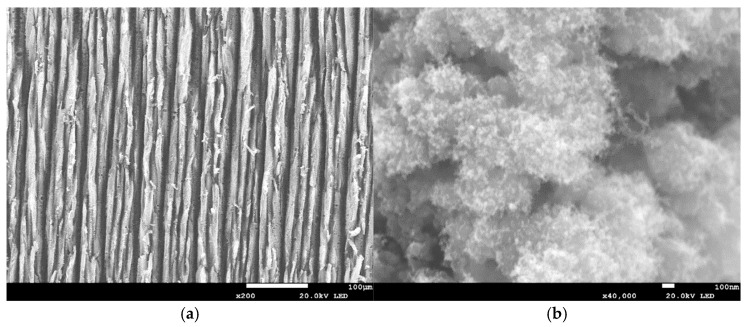
SEM micrographs of Sample 10. (**a**) Laser scan pattern with deeper valleys. (**b**) Sponge-like porous structure at high magnification. Acquired with FE-SEM JSM-7100F (own data).

**Figure 7:**
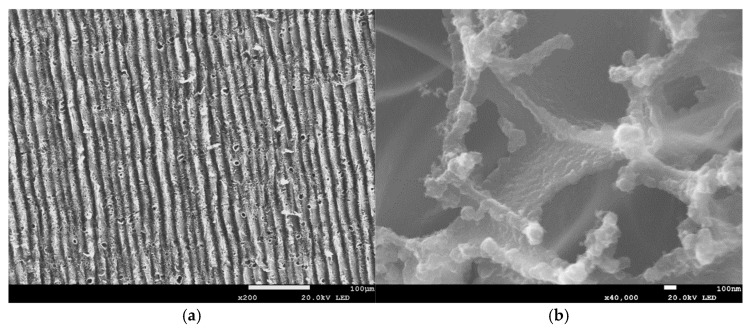
SEM micrographs of Sample 11. (**a**) Distribution of pores across the surface. (**b**) Veil-like nanostructures composed of ~100 nm agglomerations. Acquired with FE-SEM JSM-7100F (own data).

**Figure 8:**
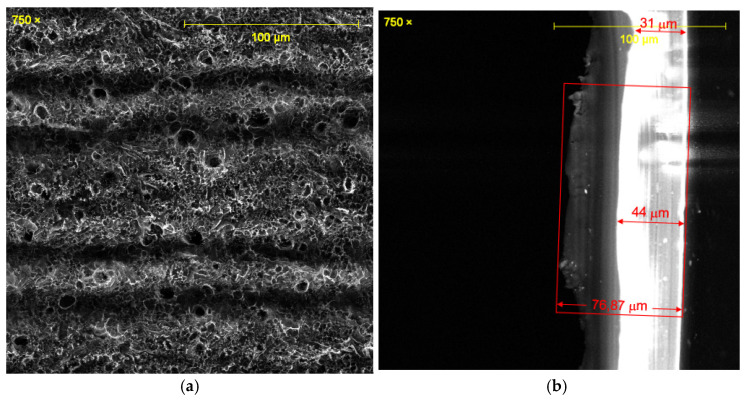
SEM micrographs of Sample 12. (**a**) Surface porosity distribution (4.4–7.6 µm). (**b**) Cross-section showing polyimide and carbonized regions. Acquired with ASPEX PSEM Express (own data).

All the laser-irradiated samples showed successful conversion of the polyimide surface into a porous, conductive carbon layer consistent with graphene or graphitic carbon. Visually, the laser-written areas turned matte black, indicating carbonization, while the surrounding polyimide remained amber-colored. Under the SEM, the LIG films exhibited a foam-like morphology comprising a network of carbon flakes and voids. Figure 2, Figure 3, Figure 4, Figure 5, Figure 6, Figure 7 and Figure 8 in the paper (SEM micrographs for Samples 1, 5, 7, 9, 10, 11, 12) illustrate the microstructural differences caused by varying laser parameters.

**Sample 1**

At 0.33 W, laser irradiation produces continuous scan tracks with near-uniform spacing and a foam-like porous carbon network (Figure 7a). High-magnification SEM further reveals micro/mesopores of ~200–500 nm (Figure 7b), indicative of multilayer, porous LIG typical of low-fluence processing. This condition serves as the baseline morphology: thinner films, finer pores, and fewer flaky features than at higher powers.

**Sample 5**

At 0.44 W, the laser scans produced evenly spaced tracks with good line uniformity (Figure 8a). SEM imaging revealed pores ranging from 150 to 1600 nm in diameter (Figure 8b), highlighting the heterogeneous and less planar structure typical of medium-power processing. The increased pore diversity at this condition suggests enhanced surface roughness, which may impact conductivity and mechanical stability.

**Sample 7**

At 0.44 W and 51 mm focus, the laser scans showed consistent spacing with reduced distance between peaks and valleys (Figure 4a). SEM revealed pores distributed randomly, with diameters ranging from ~550 to 1400 nm (Figure 4b). This morphology indicates a more homogeneous porous network compared to lower powers, contributing to improved sensor sensitivity.

**Sample 9**

At 0.55 W and 50 mm focus, the graphene patterns were well defined, with clear laser tracks visible on the polyimide surface (Figure 5a). Higher magnification revealed non-uniform regions with agglomerations at the peaks and larger pores in adjacent areas, ranging from ~1500 down to 140 nm (Figure 5b). These features are characteristic of “veil-like” morphologies formed by high-fluence irradiation, resulting in porous carbon frameworks with nanometric particles.

**Sample 10**

At 0.55 W with two passes at 50 mm focus, the laser scans of Sample 10 showed deeper valleys and more pronounced peaks (Figure 6a). High magnification revealed that the peaks consisted of circular particles of ~290–360 nm, arranged into sponge-like porous substructures (Figure 6b). The absence of veil-like morphologies compared to previous samples suggests over-carbonization, with localized particle agglomeration dominating the structure.

**Sample 11**

At 0.55 W with one pass at 51 mm focus, Sample 11 exhibited randomly distributed pores of 3.6–6.1 µm across the surface, with sharper line definition compared to Sample 7 (Figure 7a). At higher magnification, veil-like structures were observed, formed by agglomerations of ~100 nm circular particles (Figure 7b). This morphology indicates that single-pass processing at high power preserves both micro- and nanoscale porosity, enhancing surface area while avoiding over-carbonization.

**Sample 12**

At 0.55 W with two passes, Sample 12 presented randomly distributed pores of 4.4–7.6 µm, consistent with the highly porous structures observed in Samples 7 and 11 (Figure 8a). The cross-sectional analysis confirmed a total thickness of ~76.9 µm, with two distinct regions: ~31 µm of unaffected polyimide and ~44 µm of carbonized material (Figure 8b). The double-pass process enhanced porosity but also introduced structural delamination in some areas, reflecting the trade-off between increased fluence and film integrity.

In general, higher laser power and multiple passes produced a more expanded, flaky structure with larger pores between carbon structures, whereas lower power tended to yield thinner, less porous films. For example, at 0.33 W (Sample 1), the SEM images showed a relatively smooth surface with fine carbon grains and some insulating polymer matrix remaining visible. In contrast, at 0.55 W (Sample 9), the graphene had a much coarser texture, with clear ridges and folds where the laser had ablated material and left behind a 3D carbon scaffold. The distance between parallel scan lines was measured from SEM images: for a focal distance of 50 mm, the scans were well-defined and separate, whereas at 51 mm focus the lines overlapped slightly due to the larger spot size. This is consistent with the observation that Sample 5 (50 mm focus) showed distinct ridges ~70 µm apart, while Sample 7 (51 mm focus) had a more merged structure with ~50 µm peak-to-peak distance. Notably, the focal distance effect on morphology was subtle—both produced porous graphene, but the perfectly focused condition yielded slightly sharper, more pronounced graphitic ridges.

**Figure 9:**
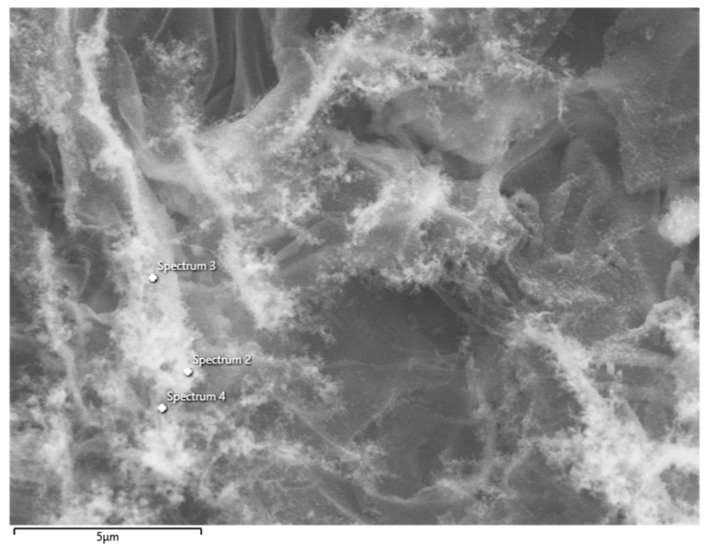
EDS analysis points for sample 9. (Results from FE-SEM JSM-7100F, EDS mode, Oxford Instruments).

The EDS analysis of Sample 9 confirmed that the laser-induced material is overwhelmingly carbon. At two of the analyzed points, the spectrum showed ~100 at. % carbon (no other elements detected above noise), and at one point a small oxygen signal (~3.9 at.%) was present. The elemental composition results in Table 2 thus indicate ~96–100% carbon with at most a few percent of oxygen locally. The EDS analysis points for sample 9 can be seen in Figure 9.

This residual oxygen likely comes from incomplete carbonization of the polyimide (which contains oxygen in its chemical structure) or surface oxidation. No metal impurities were detected, as expected since the laser and substrate were metal-free. These findings corroborate that the polyimide was successfully carbonized into a mostly carbon product (graphene/graphitic carbon). The porous nature of the LIG (as seen in SEM) means that some oxygen functionality or adsorbates might remain on internal surfaces, but overall the material can be considered a form of few-layer graphene foam.

One interesting morphological observation was the presence of micro-cracks or delamination in samples made with two passes. For instance, Sample 12 (0.55 W, 2 passes) showed some areas where the top carbon layer started to peel or separate, likely because the second laser-pass overheated regions that were already carbonized, causing them to lift. This was not catastrophic (the sensor remained electrically conductive), but it suggests that beyond a certain energy input, the benefit of additional passes diminishes and may even harm the film continuity. In contrast, single-pass samples did not show such damage; their morphology was uniform within each scan line and well adhered.

Overall, the SEM/EDS results revealed that using a 450 nm diode laser at ~0.5 W can produce a multilayer graphene-like material on polyimide, with a structure of interconnected carbon fragments. As expected, higher laser fluence (power × passes) leads to more complete carbonization (hence lower oxygen content and thicker graphene regions) at the cost of increased porosity and some loss of uniformity. Lower fluence yields thinner graphene with possibly some polymer left unconverted (raising resistance). The focus position mainly affects the sharpness of the patterned lines but has a minor effect on carbonization extent compared to power.

**Raman Spectral Analysis**

Raman spectroscopy confirmed the formation of laser-induced graphene (LIG) and enabled comparison of defect densities across samples: the characteristic D (~1350 cm^−1^), G (~1580 cm^−1^), and 2D (~2690 cm^−1^) bands were observed. Ratios of I2D/IG ≈ 0.4–0.6 and ID/IG ∼ 1 indicate few-layer graphene with substantial disorder, consistent with porous LIG; ID/ID′ ∼ 3–4 suggests defects dominated by vacancies/functionalization together with a high edge density, and a weak D′ band. Two laser passes slightly increased defects, whereas higher laser power tended to reduce ID/IG (indicative of larger graphitic domains or partial annealing); focus had a minimal effect. Overall, the samples exhibit few-layer LIG with comparable defect densities and modest improvements in order at moderate power.

**Raman spectroscopy results for sample 1**

**Figure 10:**
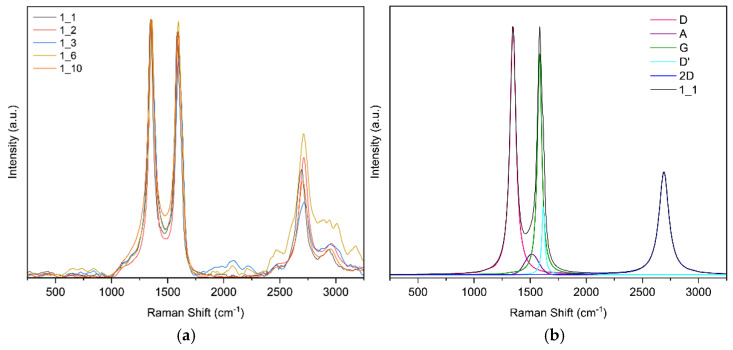
(**a**) Raman spectra of sample 1 obtained at random points and (**b**) deconvolution of the spectrum at point 1 of sample 1.

For this sample, five random points on the specimen were selected; Figure 10a shows the five curves. Figure 10b shows the band fit for point 1. The fit converged in 25 iterations with an R^2^ of 0.9870. To assess the uniformity of the samples, three additional spectra were deconvolved; the results are presented in Table 3.

**Raman spectroscopy results for sample 5**

**Figure 11:**
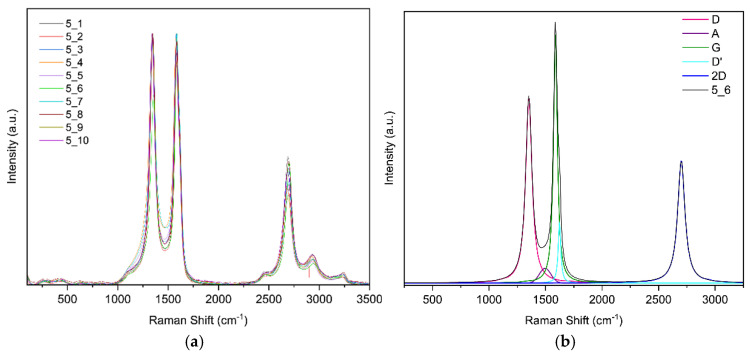
(**a**) Raman spectra of sample 5 obtained at random points and (**b**) deconvolution of the spectrum at point 6 of sample 5.

For this sample, ten random points were selected; Figure 11a shows the ten curves. Figure 11b shows the band fit. The fit converged in 21 iterations with an R^2^ of 0.9869.

**Raman spectroscopy results for sample 7**

**Figure 12:**
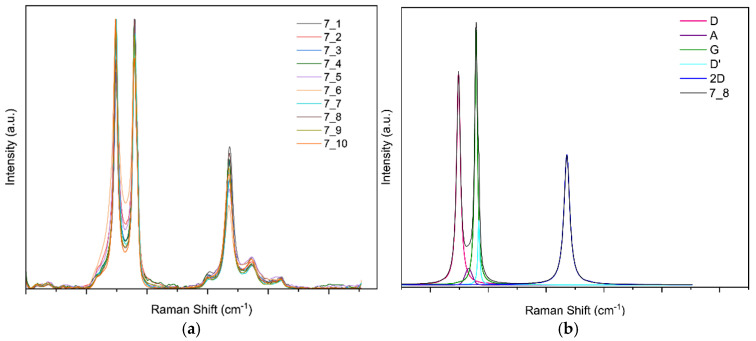
(**a**) Raman spectra of sample 7 obtained at random points and (**b**) deconvolution of the spectrum at point 8 of sample 7.

For this sample, ten random points were selected; Figure 12a shows the ten curves. Figure 12b shows the band fit. The fit converged in 31 iterations with an R^2^ of 0.9877.

**Raman spectroscopy results for sample 9**

**Figure 13:**
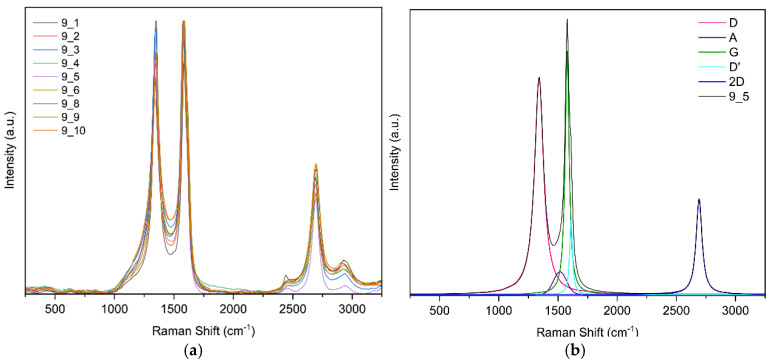
(**a**) Raman spectra of sample 9 obtained at random points and (**b**) deconvolution of the spectrum at point 5 of sample 9.

For this sample, ten random points were selected; Figure 13a shows the ten curves. Figure 13b shows the band fit. The fit converged in 25 iterations with an R^2^ of 0.9913.

**Raman spectroscopy results for sample 11**

**Figure 14:**
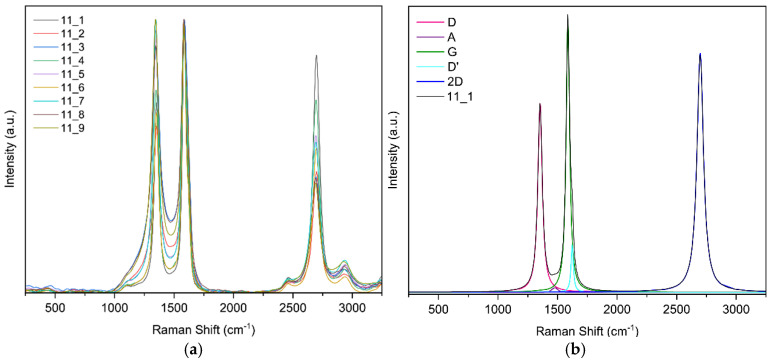
(**a**) Raman spectra of sample 11 obtained at random points and (**b**) deconvolution of the spectrum at point 1 of sample 11.

For this sample, nine random points were selected; Figure 14a shows the nine curves. Figure 14b shows the band fit. The fit converged in 11 iterations with an R^2^ of 0.9889.

**Raman spectroscopy results for sample 12**

**Figure 15:**
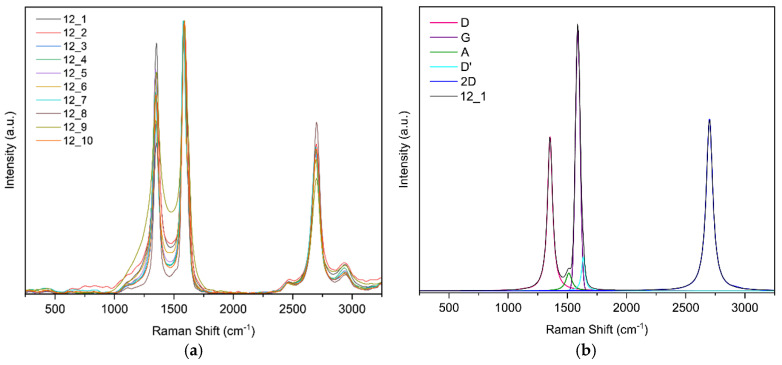
(**a**) Raman spectra of sample 12 obtained at random points and (**b**) deconvolution of the spectrum at point 1 of sample 12.

For this sample, ten random points were selected; Figure 15a shows the ten curves. Figure 15b shows the band fit. The fit converged in 17 iterations with an R^2^ of 0.9922.

**Figure 16:**
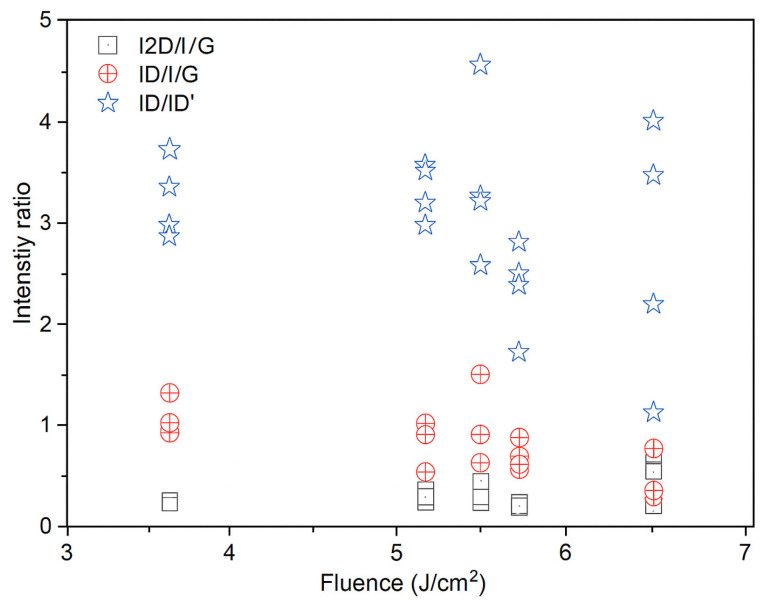
Evolution of the Raman intensity ratio vs. Fluence.

In Figure 16 you can see the evolution of the Raman intensity ratio vs. fluence.

In summary, Raman spectroscopy confirms that the laser processing converted the polyimide into a graphene-like carbon with significant disorder and multilayer characteristics. All samples are in the regime of “nanocrystalline graphite” or “turbostratic graphene,” as evidenced by the prominent D bands. The data suggest that using one laser pass at moderate power yields slightly better graphene (lower defects, possibly fewer layers) than using two passes or very low power. These findings are consistent with other LIG studies which report I_D_/I_G_ ∼1 and I_2D_/I_G_ ∼0.5 for LIG made in air without additional purification. Notably, despite the disorder, the Raman spectra of our diode-laser graphene closely resemble those of CO_2_-laser graphene from literature, indicating that the fundamental nature of LIG is retained even with the shorter wavelength laser.

**Electrical Properties (Sheet Resistance)**

**Figure 17:**
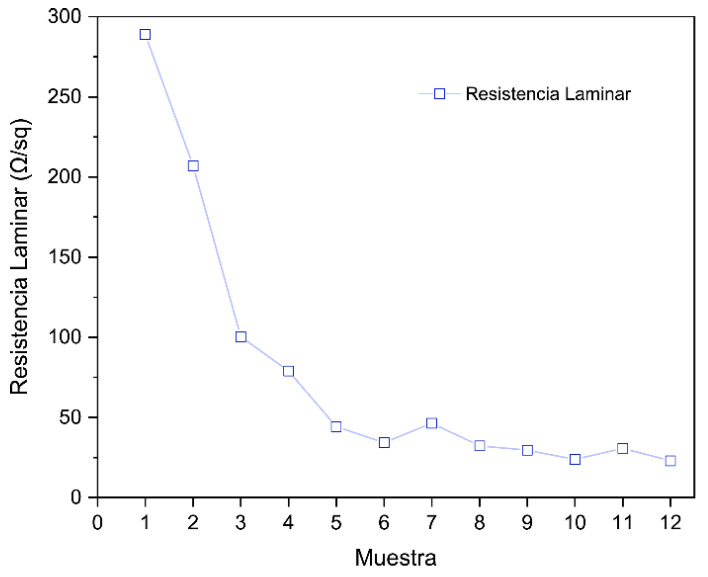
Sheet resistance per sample.

The van der Pauw measurements yielded sheet resistance values (R_S_) for each sample, which reflect the bulk conductivity of the LIG films. As expected, there was a clear dependence of sheet resistance on the laser parameters, largely governed by the laser fluence delivered. Figure 17 in the paper plots R_S_ for Samples 1–12.

In LIG, the trend is clear: increasing laser power lowers the sheet resistance (R_8_) because more polymer is converted to graphene and graphitic domains become better interconnected. With 1 pass and a 50 mm focus, Sample 1 (0.33 W) showed R_8_ in the hundreds of Ω sq^−1^, Sample 5 (0.44 W) was lower, and Sample 9 (0.55 W) dropped to tens of Ω sq^−1^. A second pass also reduces R_8_ by providing extra heating/carbonization; its effect is smaller than increasing power but can partially compensate for low power (e.g., Sample 4: 0.33 W and 2 passes < Sample 3: 0.33 W and 1 pass). At the highest fluence (0.55 W, 2 passes), R_8_ stabilizes around 20–30 Ω sq^−1^ in air, suggesting an asymptotic limit due to increased porosity or discontinuities; going lower typically requires inert atmospheres or post-treatments. Focus had minimal direct impact: Samples 5 vs. 7 (same power, 1 pass, different focus) yielded very similar values (~60 vs. ~55 Ω sq^−1^), with differences within experimental error. Overall, conductivity is tunable via laser parameters and is dominated by power (total delivered energy), achieving ≈20–30 sq^−1^ at high fluence and higher values at lower powers, comparable to or better than those reported in the literature [20].

**Strain Sensing Performance**

**Figure 18:**
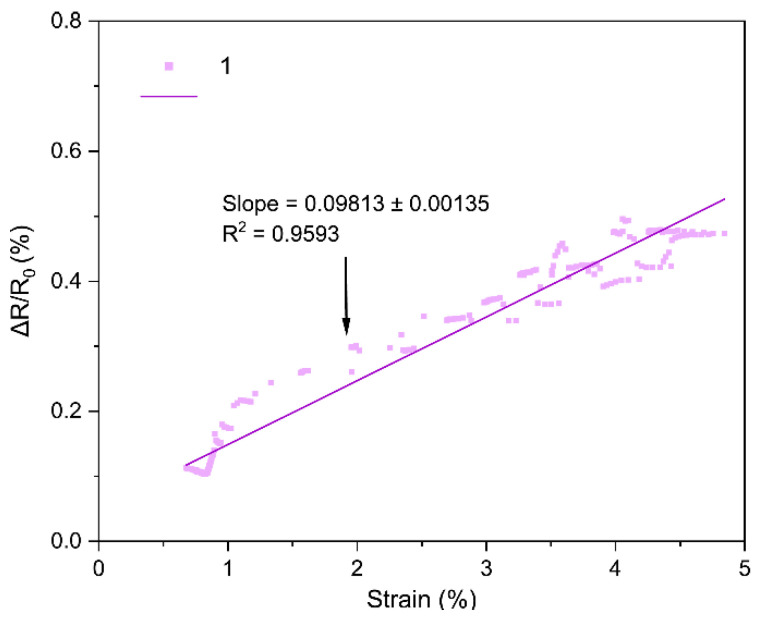
Normalized resistance vs. three-point bending strain, sample 1.

**Figure 19:**
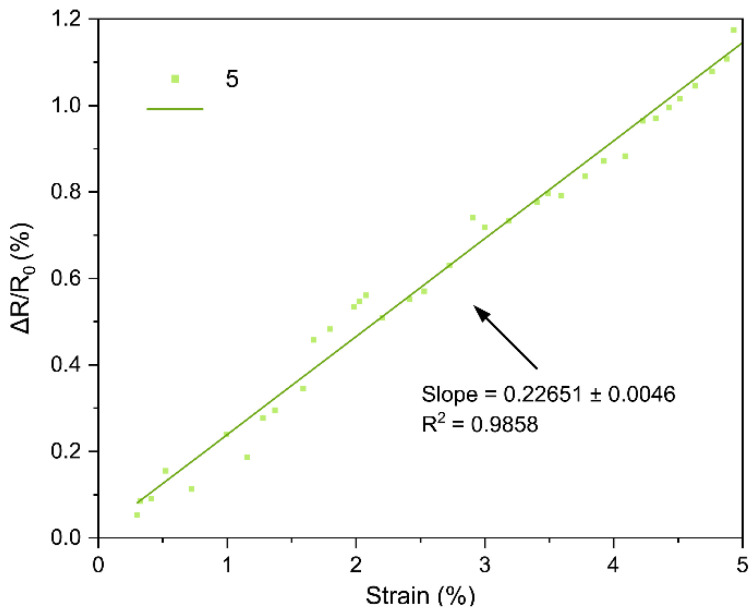
Normalized resistance vs. three-point bending strain, sample 5.

**Figure 20:**
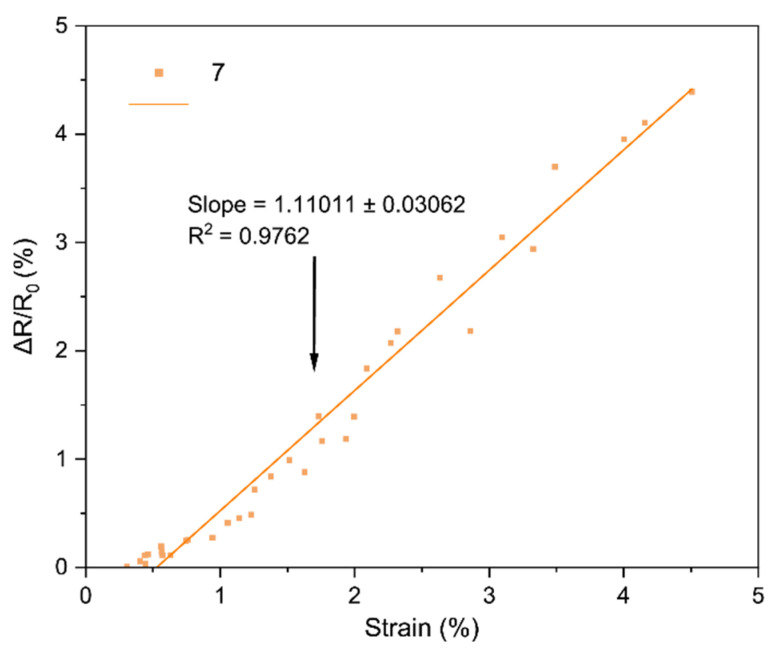
Normalized resistance vs. three-point bending strain, sample 7.

**Figure 21:**
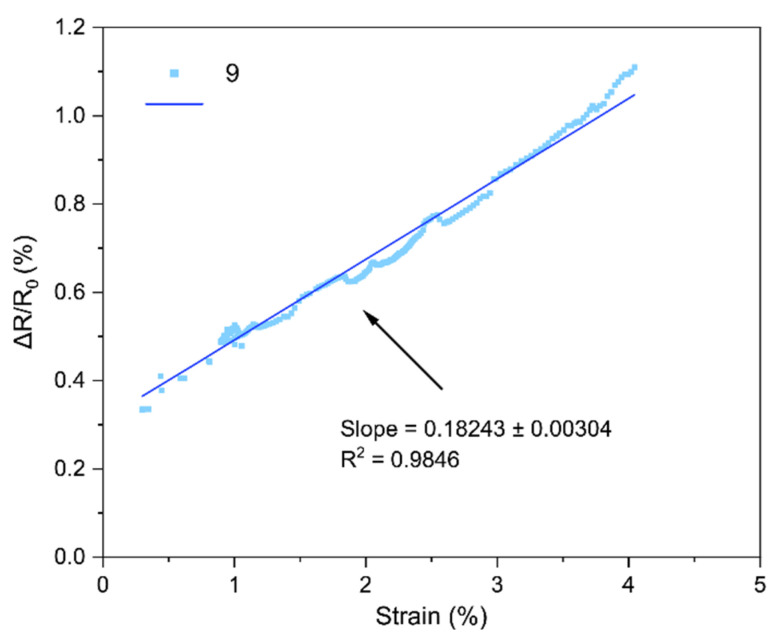
Normalized resistance vs. three-point bending strain, sample 9.

**Figure 22:**
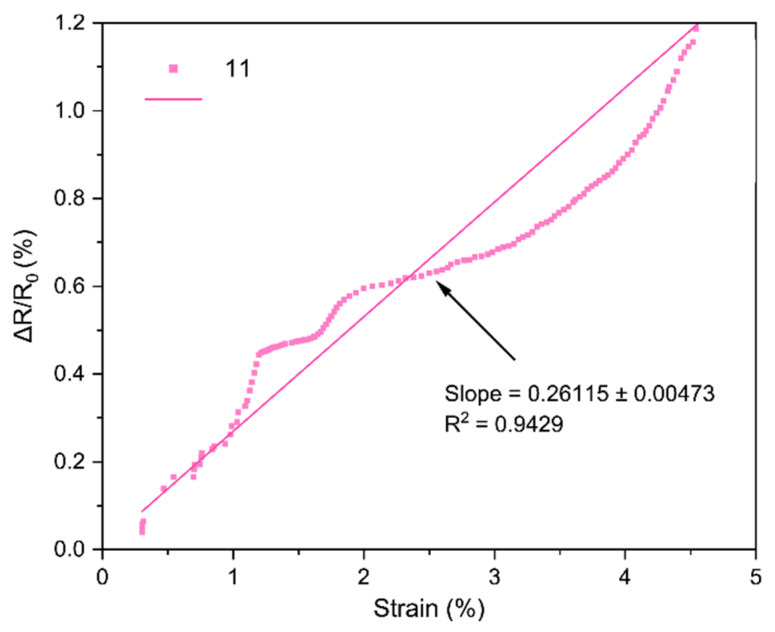
Normalized resistance vs. three-point bending strain, sample 11.

**Figure 23:**
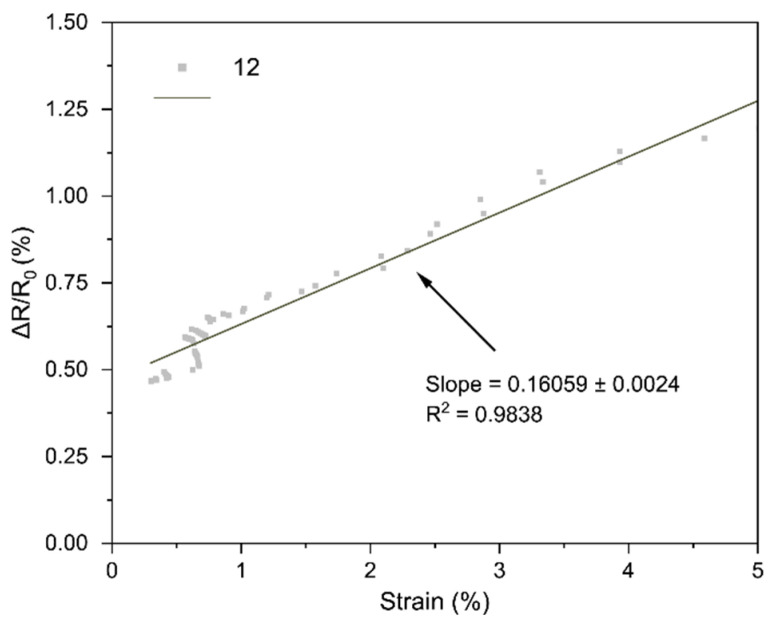
Normalized resistance vs. three-point bending strain, sample 12.

The core performance of the LIG strain sensors is encapsulated by their strain gauge factor (GF), which we extracted from the bending tests. Figure 18, Figure 19, Figure 20, Figure 21, Figure 22 and Figure 23 in the paper show the normalized resistance vs. strain curves for Samples 1, 5, 7, 9, 11, 12, from which the GF slopes were obtained.

**A. GF curve from the three-point bending test of Sample 1**

The slope of the curve in Figure 18 represents the strain gauge factor of sensor sample 1; the value is 0.09813 ± 0.00135 with R^2^ = 0.959 for strain values from 0 to 5%.

**B. GF curve from the three-point bending test of Sample 5**

The slope of the curve in Figure 19 represents the strain gauge factor of sensor sample 5; the value is 0.22651 ± 0.0046 with R^2^ = 0.9858 for strain values from 0 to 5%.

**C. GF curve from the three-point bending test of Sample 7**

The slope of the curve in Figure 20 represents the strain gauge factor of sensor sample 7; the value is 1.11011 ± 0.03062 with R^2^ = 0.9762 for strain values from 0 to 4.5%.

**D. GF curve from the three-point bending test of Sample 9**

The slope of the curve in Figure 21 represents the strain gauge factor of sensor sample 9; the value is 0.18243 ± 0.00304 with R^2^ = 0.9846 for strain values from 0 to 4%.

**E. GF curve from the three-point bending test of Sample 11**

The slope of the curve in Figure 22 represents the strain gauge factor of sensor sample 11; the value is 0.26115 ± 0.00473 with R^2^ = 0.9429 for strain values from 0 to 4.5%.

**F. GF curve from the three-point bending test of Sample 12**

The slope of the curve in Figure 23 represents the strain gauge factor of sensor sample 12; the value is 0.16059 ± 0.0024 with R^2^ = 0.9838 for strain values from 0 to 5%.

All sensors showed an approximately linear increase in resistance under tensile strain (with no appreciable hysteresis up to ~4–5% and R^2^ = 0.94–0.99), indicating good LIG adhesion. Gauge factors (GF) ranged from ~0.1 to ~1.11, with Sample 7 (GF ≈ 1.11 ± 0.03) being the most sensitive; Sample 1 (lowest power) had GF ≈ 0.10. In general, increasing power from 0.33 to 0.44 W raised GF, but at 0.55 W it decreased, suggesting an intermediate optimal power: moderate power yields a conductive network with microcracks/junctions that open under strain (larger ΔR), whereas too low a power produces poorly conducting paths and too high a power forms robust films with lower sensitivity. Two passes reduced GF relative to one pass at the same power (e.g., at 0.55 W: ~0.16 vs. ~0.26), consistent with the typical trade-off between higher conductivity and lower sensitivity in percolative networks. Focus had a minor effect, though 51 mm tended to give slightly higher GF than 50 mm, possibly due to more strain-responsive microdefects. Compared with ambient-condition literature, our GFs (≈0.2–1.1) are within range and could be increased using stretchable substrates, encapsulants, or microstructures; nevertheless, the observed linearity and stability are adequate for practical flexion/deformation monitoring.

## 4. Discussion

The investigation of conventional methods for obtaining graphene faces the challenge of using toxic chemicals, which represents a significant risk to the environment [21]. Graphene obtained through laser synthesis has attracted interest in research for its potential to overcome barriers to the study and large-scale production of graphene. In particular, the use of diode lasers has emerged as a highly effective and economical alternative for graphene production. This methodology offers significant advantages, such as the rapid fabrication of high-quality graphene, reduced costs, and the ability to employ a variety of precursors ranging from polymers to cellulose-based materials [22,23]. Research on graphene obtained with lasers presents several relevant aspects that must be studied and tested to refine the method. These aspects include the selection of the laser, substrate, synthesis parameters, whether graphene transfer to another material is required, and the intended application [24,25]. In this research, the influence of laser power, focal distance, and number of scans of a 450 nm diode laser was studied.

SEM micrographs showed that pore sizes tend to decrease as laser power increases. According to Wang et al. [26], the reason for this phenomenon is that as the laser energy increases, the irradiated area undergoes greater evaporation or decomposition [26]. It was also observed that there is a wide range of pore sizes, apparently related to the non-uniformity of the raster caused by laser movement due to the servomotors. In general, SEM revealed that the material exhibits a foam-like appearance, with a hierarchical porous structure organized at different levels, i.e., pores are present in different sizes and distributed in an orderly manner [27].

In Raman spectroscopy, the characteristic 2D band of graphene-based materials was observed. The intensity ratios are shown graphically in Figure 21 where it was noted that the D-to-G band intensity ratio tends to decrease as laser fluence increases. According to Esqueda-Barron et al. [28], an increase in this value indicates a higher number of defects and a reduction in the crystallinity of the sample [28]. Laser irradiation causes localized phase transformations such as melting, vaporization, and even recrystallization, which may increase defects, reflected in the I_D_/I_G_ ratio [21]. This can be explained by the fact that when the laser irradiates the precursor material containing graphene, sp^2^ rings are lost, and the intensity of the D band increases [29]. The 2D-to-G band intensity ratio tended to increase, providing information about the number of graphene layers in the sample. In this case, most values ranged between 0 and 1, indicating multilayer graphene [30]. The D-to-D′ band intensity ratio was mostly between 3 and 4, revealing that edge-type defects predominate [31], which is consistent with SEM observations showing that the samples are highly porous and present a large number of edges. Defects increased as laser energy also increased, consistent with the study of Huang et al. [32], where increasing energy likewise increased defects in their samples [32]. One data point showed this ratio rising to 4.5, indicating low structural disorder and that defects were mainly a mixture of vacancy-type defects and edge defects [28]. Meanwhile, according to Venezuela et al., values approaching 1.3 correspond to point defects, which occur when an atom is missing or occupies a position different from the ideal in the crystal lattice [33].

The calculated standard error values of the intensity ratios showed relatively low variability, up to 12.8% in the 2D-to-G ratio, indicating that several points on a sample are moderately homogeneous in terms of the number of graphene layers. With respect to the I_D_/I_G_ and I_D_/I_D′_ ratios, the standard error reached up to 19.7% and 58.4%, respectively, demonstrating that the samples are not very homogeneous. This reduced homogeneity may be caused by the limited precision of the servomotors at the microscopic level, by the high percentage of overlap between laser scans.

The Raman spectra of the analyzed samples presented better results than those reported by Yen et al., where the 2D-to-G intensity ratios fluctuated between 0.41 and 0.50 [14]. In the present study, values ranged from 0.39 to 0.99, demonstrating that even without using a more advanced CO_2_ laser, it is possible to obtain graphene with fewer layers.

The Van der Pauw sheet resistance measurements revealed a decreasing trend in values with increasing fluence. However, a reduction in sheet resistance was observed when two scans were performed at the same power and focus. Moreover, sheet resistance values appeared to converge toward approximately 20 Ω sq^−1^ as fluence increased. Consequently, it can be concluded that under the conditions evaluated in this study, sheet resistance values do not fall below this level. This decrease in resistance is consistent with SEM observations, since smaller pore sizes increase the material’s specific surface area, which in turn reduces resistance to the flow of electrical current. These results contrast with those of Wang et al. [26], where, using a UV laser, the authors obtained a minimum sheet resistance of ~150 Ω sq^−1^, while using a CO_2_ laser reduced sheet resistance to the levels achieved in this study [26].

Regarding the strain gauge factor, the results showed a satisfactory fit to the bending test data, with R^2^ values ranging from 0.9429 to 0.9858. These results indicate that the gauge factor values, representing the slope of the normalized resistance vs. bending strain plots, adequately fit the data from the three-point bending tests. The gauge factor values are shown in Table 4, where they are consistent with the study of Liu et al. [18], who reported gauge factor values up to 1 at deformation levels of 4.5–5% using CO_2_ laser powers up to 0.75 W under ambient conditions on polyimide [18]. The results contrast with other studies; for instance, Huang et al. [32] obtained a gauge factor of ~37.8 with a 40 W CO_2_ laser irradiating polyimide. In that case, the strain gauge factor was measured at strains up to 31.8%, and the main difference lies in the use of a silicone rubber coating and a sensor geometry six times longer than in this work [32]. As noted during the geometry selection, elongated patterns were discarded because they caused deformation of the polyimide, and once the carbonized material deformed, it detached from the substrate. In summary, one of the main differences between the gauge factor values of that study and Huang et al. [32] is the use of flexible coatings, which allow the fabrication of more complex geometries and also enhance the gauge factor values of the sensors [16].

Regarding the gauge factor values and the initial fabrication parameters, it can be observed that between samples 1 and 5 there is a notable improvement due to the increase in laser power. Between samples 5 and 7 or 9 and 11, the gauge factor improved as the focal distance increased. Finally, in the comparison between samples 11 and 12, the gauge factor decreased when two perpendicular scans were performed on the same geometry, possibly due to the reduction of graphene caused by further carbonization of the material during the second scan.

Sample 7 stands out as the best in terms of three-point bending response, as it presented the highest gauge factor value. This factor is considered a measure of sensitivity for this type of sensor [34]; therefore, it can be stated that the sensor is more sensitive when the gauge factor value is higher. As shown in Table 5.

The results regarding laser fluence reveal that, despite considering the focal distance variable, the predominant factor in calculating fluence is laser power. This indicates that power is the main determinant of the amount of energy delivered to the material per unit area, while focal distance has a secondary influence in comparison. As mentioned in the study by Choi et al., the effect of focal distance can be observed in the morphology of the samples [35].

The influence of focal distance was analyzed by comparing samples 5 and 7, which had the same fabrication parameters except for focal distance. SEM revealed that at a focal distance of 50 mm (sample 5), the distance between peaks and valleys was greater. This is logical, since the laser manufacturer specifies that the laser is best focused at 50 mm, resulting in higher resolution [36]. Regarding Raman analyses, the I_2D_/I_G_ ratio at 50 mm was slightly higher, indicating fewer graphene layers as the laser focus improves. With respect to the I_D_/I_G_ and I_D_/I_D′_ ratios, values tended to increase when the laser was focused at 51 mm, suggesting a higher number of defects. However, the differences in intensity ratios were minimal, as shown in Table 3.

The primary objective of this research was to assess the feasibility of producing laser-induced graphene (LIG) using a low-power commercial diode laser and to validate its functionality in strain sensing. The experimental results confirmed that laser power is the dominant factor influencing the structural and electrical performance of the material, with intermediate power conditions yielding the highest gauge factors. While focal distance and the number of passes had secondary effects, they also influenced microstructural uniformity and defect density. Importantly, this study demonstrates that functional LIG with strain-sensing capability can be achieved using an accessible, low-cost diode laser, in contrast to the conventional reliance on high-power CO_2_ systems. Beyond validating the fabrication method, these findings open opportunities for integrating diode-laser LIG into flexible, sustainable, and affordable devices, with potential applications in structural health monitoring, wearable electronics, and next-generation environmentally friendly graphene-based sensors.

## 5. Conclusions

A bending sensor prototype was successfully fabricated by irradiating a polyimide sheet with a commercial laser with a wavelength of 450 nm. The electrodes obtained, according to the results shown, are composed of a graphene-type, porous structure with low laminar electrical resistance and a gauge factor of 1.11 for deformations up to 5%.

The effect of laser power, focal distance, and number of scans on the performance of the bending sensors was studied. The material characterization results demonstrated that low-cost graphene was indeed obtained, and its characteristics changed depending on the studied variables. Laser power is the key factor when manufacturing bending sensors from graphene. The focal distance parameter did not have a significant impact on the results of laser-induced graphene fabrication. Additionally, increasing the number of scans decreases the quality of the material obtained and the sensor’s gauge factor.

SEM micrographs and Raman spectroscopy showed that the samples were not homogeneous at the microscopic level. The standard error values in the intensity ratios showed low variability in the number of graphene layers but a higher error in the homogeneity of the samples in the I_D_/I_G_ and I_D_/I_D’_ ratios, possibly due to the low precision of the servomotors and overlap in the laser scans.

In this study, lower laminar resistances were obtained compared to those reported in previous research. Concurrently, a directly proportional relationship was observed between pore sizes and the laminar resistance of the samples. However, it cannot be concluded that the gauge factor values show a very marked trend based on the mentioned pore sizes, due to other factors such as poor microscopic homogeneity of the samples, overlaps between scans, or the lack of samples with the same manufacturing parameters.

The graphene obtained in this work is low-cost and allows for varied geometries. Furthermore, the Raman spectra of the samples show significant similarity to other works where graphene was obtained using CO_2_ lasers, and it is even possible to improve the results through proper selection of parameters for obtaining graphene with the commercial 450 nm-wavelength laser.

In the context of laser-induced graphene synthesis, it is important to clearly understand the specific application intended for the graphene obtained by this method. In this sense, it is concluded that there is no perfect method for obtaining graphene; rather, it is essential to find a balance between the desired goals and the available resources. Additionally, in terms of material acquisition parameters, the correct selection of the geometry required for the application is of vital importance, an aspect that was successfully achieved within the scope of this research.

It is essential to conduct future research focused on analyzing the influence of various manufacturing parameters, atmosphere types, alternative substrates, as well as the coating of graphene-based sensors, etc., studies that use a greater number of samples in order to expand the information and develop models that allow optimizing graphene production based on the specific needs of the application.

This work demonstrated that laser-induced graphene (LIG) can be fabricated using a low-power commercial diode laser (450 nm), offering a cost-effective and environmentally friendly alternative to conventional CO_2_ systems. Laser power was identified as the key parameter controlling the structural and electrical properties of LIG, with intermediate power levels (0.44 W) yielding the highest gauge factor (1.11).

The novelty of this study lies in showing that functional strain-sensing materials can be produced with accessible, low-cost equipment, thereby democratizing graphene-based technologies. These findings open opportunities for flexible and affordable strain sensors in applications such as structural health monitoring, wearable electronics, and sustainable devices.

## Figures and Tables

**Table 1:** Multi-level experimental design of this research.

Order	Power (W)	Focal Length (mm)	Number of Passes
1	0.330	50	1
2	0.33	50	2
3	0.33	51	1
4	0.33	51	2
5	0.44	50	1
6	0.44	50	2
7	0.44	51	1
8	0.44	51	2
9	0.55	50	1
10	0.55	50	2
11	0.55	51	1
12	0.55	51	2

(Own source).

**Table 2:** Elemental composition results of sample 9.

Spectrum Label	Spectrum 2	Spectrum 3	Spectrum 4
C	9606	10,000	10,000
O	394	-	-
Total	10,000	10,000	10,000

**Table 3:** Raman spectroscopy results.

Sample	Point	ID (a.u.)	IA (a.u.)	IG (a.u.)	ID’ (a.u.)	I2D (a.u.)	I2D/IG	ID/IG	ID/ID’
1	1_1	0.96	0.08	0.85	0.26	0.40	0.47	1.12	3.67
	1_2	0.97	0.07	0.88	0.29	0.42	0.48	1.11	3.36
	1_3	0.97	0.12	0.65	0.35	0.24	0.37	1.49	2.79
	1_10	0.93	0.13	0.76	0.32	0.34	0.45	1.22	2.87
	σx	0.011	0.016	0.051	0.019	0.040	0.025	0.087	0.208
5	5_1	0.97	0.06	0.85	0.27	0.48	0.57	1.14	3.62
	5_2	0.97	0.05	0.81	0.27	0.38	0.47	1.19	3.56
	5_6	0.71	0.06	0.95	0.23	0.47	0.49	0.75	3.07
	5_8	0.97	0.06	0.88	0.30	0.46	0.53	1.10	3.28
	σx	0.065	0.002	0.029	0.013	0.023	0.022	0.101	0.127
7	7_3	0.74	0.07	0.91	0.27	0.45	0.50	0.81	2.70
	7_4	0.77	0.79	0.47	0.17	0.31	0.66	1.66	4.57
	7_7	0.96	0.07	0.87	0.29	0.39	0.45	1.10	3.34
	7_8	0.79	0.06	0.95	0.24	0.49	0.51	0.83	3.30
	σx	0.049	0.180	0.113	0.026	0.040	0.044	0.197	0.391
9	9_3	0.88	0.12	0.83	0.35	0.35	0.42	1.06	2.52
	9_4	0.70	0.09	0.89	0.27	0.40	0.45	0.79	2.62
	9_5	0.79	0.08	0.89	0.27	0.35	0.39	0.89	2.91
	9_6	0.63	0.13	0.77	0.33	0.33	0.43	0.82	1.88
	σx	0.055	0.012	0.029	0.021	0.014	0.012	0.060	0.216
11	11_1	0.69	0.02	0.99	0.17	0.87	0.88	0.69	4.01
	11_2	0.54	0.07	0.92	0.23	0.42	0.46	0.59	2.41
	11_4	0.76	0.02	0.72	0.54	0.71	0.99	1.05	1.40
	11_6	0.64	0.03	0.99	0.18	0.53	0.53	0.65	3.52
	σx	0.045	0.013	0.063	0.088	0.099	0.128	0.104	0.584
12	12_3	0.59	0.04	0.98	0.17	0.51	0.52	0.60	3.51
	12_6	0.58	0.06	0.96	0.19	0.48	0.50	0.61	3.06
	12_7	0.72	0.03	0.97	0.22	0.53	0.54	0.74	3.31
	12_8	0.56	0.07	0.95	0.12	0.63	0.66	0.59	4.54
	σx	0.035	0.009	0.005	0.019	0.033	0.036	0.035	0.327

(Own source).

**Table 4:** Strain Gauge Factor values obtained in the graphene sensors.

Sample	Strain Gauge Factor
1	0.0981
5	0.2265
7	1.1101
9	0.1824
11	0.2612
12	0.1606

**Table 5:** Strain Gauge Factor values obtained in the graphene sensors. Summary of DOE conditions and main results for LIG sensors.

Sample ID	Laser Power (W)	Focal Distance (mm)	Passes	Gauge Factor (GF)	Notes/Key Observations
1	0.33	50	1	0.098	Baseline, low power
5	0.44	50	1	0.227	Improvement due to higher power
7	0.44	51	1	1.110	Best GF, high sensitivity
9	0.55	50	1	0.182	Higher power, stable response
11	0.55	51	1	0.261	Moderate GF
12	0.55	51	2	0.161	Double pass reduced performance

## Data Availability

Data are contained within the article.
